# A Green Method for Processing Polymers using Dense Gas Technology

**DOI:** 10.3390/ma3053188

**Published:** 2010-05-11

**Authors:** Roshan B. Yoganathan, Raffaella Mammucari, Neil R. Foster

**Affiliations:** 1 Department of Pharmaceutical Sciences, Leslie Dan Faculty of Pharmacy, University of Toronto, 144 College Street, Toronto, Ontario M5S 3M2, Canada; 2 School of Chemical Engineering, Chemical Sciences Building, University of New South Wales, Sydney NSW 2052, Australia

**Keywords:** dense gas technology, polymer processing, green technology, biomedical polymers, drug delivery system, polymer blends, polymerization, polycarbonate, polycaprolactone, ibuprofen

## Abstract

Dense CO${}_{2}$ can be used as an environmentally-benign polymer processing medium because of its liquid-like densities and gas-like mass transfer properties.In this work, polymer bio-blends of polycarbonate (PC), a biocompatible polymer, and polycaprolactone (PCL), a biodegradable polymer were prepared. Dense CO${}_{2}$ was used as a reaction medium for the melt-phase PC polymerization in the presence of dense CO${}_{2}$-swollen PCL particles and this method was used to prepare porous PC/PCL blends. To extend the applicability of dense CO${}_{2}$ to the biomedical industry and polymer blend processing, the impregnation of ibuprofen into the blend was conducted and subsequent dissolution characteristics were observed.

## 1. Introduction

### 1.1. Polycarbonates

**Figure 1:**
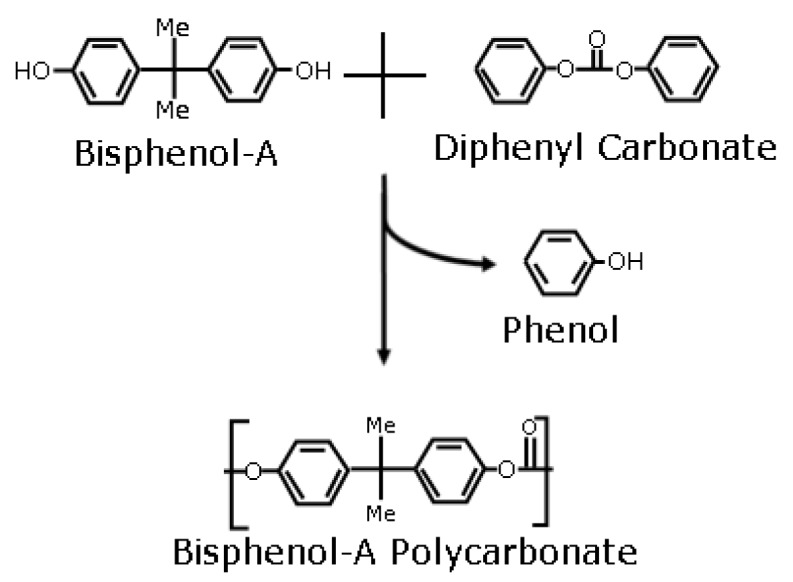
Synthesis of polycarbonate by the transesterification of bisphenol-A.

An aromatic polycarbonate (PC) such as bisphenol-A (BPA) PC, possess strong mechanical properties, heat resistance and unique optical clarity. Aromatic PC have been used in the medical field in filter cartridges for renal dialysis, blood-management products, surgical instruments, and intravenous connection components [1]. The conventional synthesis of PC involves the use of phosgene (COCl${}_{2}$), an environmentally hazardous and toxic substance. Phosgene poses an occupational risk and once released in the form of manufacturing waste, it is an environmental hazard. Acute and chronic effects of phosgene exposure can be as severe as liver failure and lung damage. Consequently, health-related and environmental regulations have been imposed on the polymer industry regarding the use of phosgene [2,3]. One phosgene-free method of PC synthesis is the transesterification of BPA and diphenyl carbonate (DPC) (Figure 1), a type of polycondensation reaction with phenol as the by-product. Few environmentally benign methods of PC synthesis involving dense CO${}_{2}$ have been documented [2,4,5,6,7].

#### Polycarbonate Synthesis in Dense CO${}_{2}$

Carbon dioxide has been used in heterogeneous polymerizations of PC such as dense gas (DG) melt-phase polymerization, solid-state polymerization (SSP) and dispersion polymerization [4,5,6]. In all cases, CO${}_{2}$ was used to extract phenol, which is highly soluble in dense CO${}_{2}$. As the phenol is extracted, the synthesis is driven towards the formation of higher molecular weight PC [8]. The transesterification of BPA and DPC was conducted in the melt phase and CO${}_{2}$ was used to extract the by-product [4,5]. In another method, Gross *et al.* reported that SSP was most effective on PC particles when using CO${}_{2}$ [5]. Particles 20 *μ*m in size underwent SSP to produce PC with weight-average molecular weight (M${}_{w}$) of 1.75 x 10${}^{4}$ Da whereas particles 3.6 mm in size produced PC of M${}_{w}$ 7.5 x 10${}^{3}$ Da. For SSP conducted in dense CO${}_{2}$ the particle size and available surface area have an effect on the M${}_{w}$ of the polymer. Decreased particle size provides a larger surface area for dense CO${}_{2}$ to interact with and extract phenol, and corresponds to higher M${}_{w}$ PC. Also, dispersion polymerization (CO${}_{2}$ as dispersing phase) produced PC nanoparticles with M${}_{w}$ 3.0 x 10${}^{5}$ Da by tuning the surfactant concentration, CO${}_{2}$ pressure and temperature [6]. In the aforementioned studies, dense CO${}_{2}$ was used successfully to synthesize PC, thereby circumventing the use of phosgene.

### 1.2. Polycaprolactone

Polycaprolactone is a well-known biodegradable polymer widely used in the biomedical field. It is commonly used for tissue engineering applications because of its favourable and prolonged rate of degradation *in vivo* [9]. Dense gas processing of PCL has been used to foam the polymer and create micro and nano-architectures in it [10,11].

### 1.3. Polymer Blends

Bio-blends (blends involving at least one biodegradable component) are gaining wide interest in the biomedical field for drug delivery devices and implantable/non-implantable formulations, and in the agricultural field for materials/packaging applications [12,13,14]. Conventional blending techniques, such as solvent casting and melt extrusion use toxic solvents or are conducted at high temperatures [15,16,17,18]. The dense gas technology (DGT) can provide polymer synthesis and polymer blend processing methods void of toxic solvents and low temperature operation. According to Tomasko et al. the next wave of dense CO${}_{2}$ polymer processing applications is in the field of polymer blends [19].

In studies reported in the literature, dense CO${}_{2}$-swollen polymers have been impregnated with monomers and subsequent polymerization reactions were conducted to produce polymer blends. Watkins *et al.* [20,21,22,23] and Li *et al.* [23] conducted the free radical polymerization of styrene in dense CO${}_{2}$-swollen polyethylene (PE) to create polystyrene (PS)/PE blends . Dense CO${}_{2}$ has been used to increase the compatibility of immiscible polymer blends, thereby creating fine dispersed domains [19,24,25].

Polymer blends of PC/PCL have potential industrial and biomedical applications both *in vivo* and *in vitro*. The biodegradability of PCL has long been exploited to culture biological components. The applicability of PCL can be extended and its mechanical properties enhanced by creating a bio-blend with a stronger polymer such as PC. The use of dense CO${}_{2}$ as a bio-blend processing medium was investigated in this work.

In some cases, PC/PCL blends have proved to be miscible over the entire composition range [26,27,28,29,30]. Dense gas technology can be used to create PC/PCL bio-blends by synthesizing PC in the presence of dense CO${}_{2}$-swollen PCL. Although, the dense CO${}_{2}$-swollen polymers are usually the major component of blends prepared by DGT, in this work PCL was the minor component.

In this study, a dense CO${}_{2}$ melt polymerization of BPA and DPC was conducted to prove the applicability of CO${}_{2}$ as a polymerization medium for the production of an aromatic PC (BPA-PC). Dense CO${}_{2}$ was also used as an extraction medium to flush out the CO${}_{2}$-soluble by-product phenol. Sodium hydroxide (NaOH), a cheap and relatively safe catalyst, was used for the dense CO${}_{2}$ melt polymerization.

Polycarbonate samples were analyzed using reverse-phase high performance liquid chromatography (RP-HPLC), electron spray ionization-mass spectrometry (ESI-MS) and gel permeation chromatography (GPC). A RP-HPLC method was developed to measure the conversion of BPA for the preliminary melt polymerization experiments. The MS technique was used to validate the PC structure for preliminary molecular weight determination. The GPC was used to obtain polymer molecular weight.The melt-phase PC synthesis was then conducted in the presence of dense CO${}_{2}$-swollen PCL particles producing porous PC/PCL blends. The impregnation of ibuprofen using dense CO${}_{2}$ was also carried out on PCL and DG processed bio-blends. Subsequent dissolution studies were used to measure the drug-release properties of the samples.

## 2. Materials and Methods

Bisphenol A (99% purity), DPC (99% purity) , NaOH (99% purity), PCL (M${}_{w}$ = 1.2 x 10${}^{4}$ Da, 99% purity) and tin(II)ethyl hexanoate (Sn(Oct${}_{2}$)) (95% purity) were used as received from Sigma Aldrich. The PCL monomer, *ε*CL (99% purity) was purchased from Fluka. Ibuprofen was supplied by Nanomaterials Technology (Singapore). Cylinders of compressed N${}_{2}$ grade 3.0 and compressed CO${}_{2}$ grade 2.5 were used as supplied by Linde.

**Figure 2:**
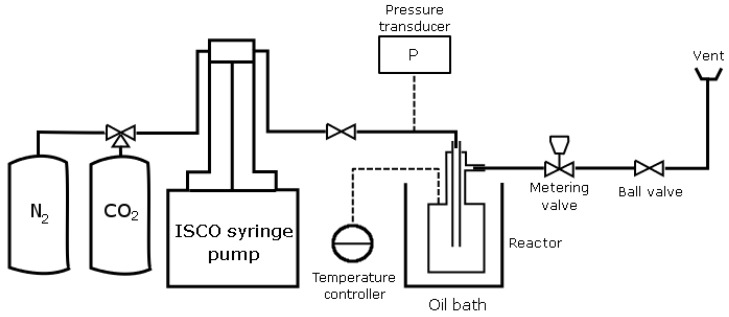
High pressure vessel setup.

Materials were loaded into a high pressure vessel with an internal volume of 10 mL as depicted in Figure 2 . The reactor was placed in a temperature-controlled oil bath and its content was magnetically stirred. Pressure and temperature were monitored by a Druck DPI 260 pressure transducer and WiseStir MSH-20D thermocouple, respectively.

The PC synthesis experiments were carried out in an inert environment (N${}_{2}$) with varying reaction times. Preliminary experiments with monomer to catalyst mole ratios of 1:1 and 2:1 caused hydrolysis reactions that inhibited the polymerization. A monomer to catalyst mass ratio of 100:1 which is equivalent to a BPA:DPC:NaOH mass ratio of 50:50:1 was used for all experiments. The operating pressure of N${}_{2}$ was 1 bar. At the end of each experiment, the reactor was cooled in cold water.

### 2.1. Two-stage PC Synthesis in N${}_{2}$ and CO${}_{2}$

Two-stage experiments were conducted under 1 bar N${}_{2}$ in an isolated reactor (Stage 1) and then flushed with a continuous flow of 200 bar dense CO${}_{2}$ (Stage 2). The continuous flow of dense CO${}_{2}$ was introduced to help extract the phenol. All experiments were conducted in the melt phase. The dense CO${}_{2}$ flow-rates were controlled using a metering valve.

### 2.2. Three-stage PC Synthesis

**Figure 3:**
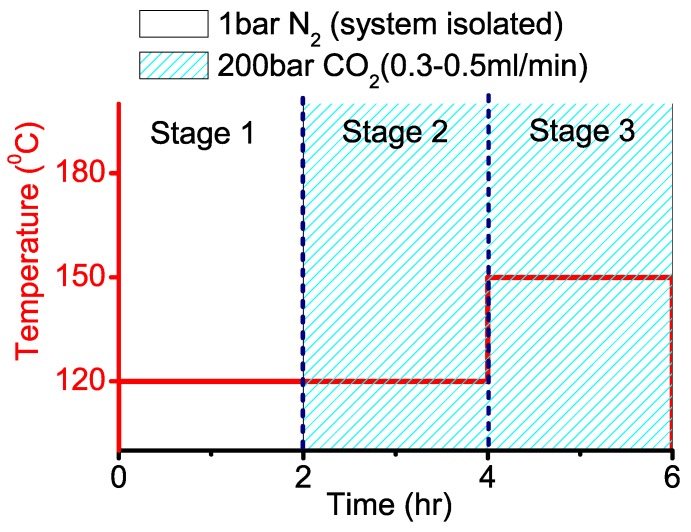
Three stages for the production of sample PC-6 hrs.

The three-stage PC synthesis experiments were conducted using the setup in Figure 2. Operating conditions depicted in Figure 3 were used.

### 2.3. Synthesis of PC With Dense CO${}_{2}$-foamed PCL

Bisphenol A, and DPC were ground together in equivalent molar ratios, and then placed in the high pressure reactor (Figure 2) with the catalyst (Table 1). Polycaprolactone from Sigma was ground separately using a mortar and pestle, and then loaded into the same 10 mL high pressure reactor. Samples were subjected to the experimental method depicted in Figure 3. All samples were analyzed using TGA, DSC, scanning electron microscopy (SEM), and BET nitrogen adsorption.

**Table 1:** Sample composition for PC synthesis in dense CO${}_{2}$-foamed PCL.

Sample	Mass Ratio
PC80/PCL20	200mg BPA : 200mg DPC : 4mg NaOH : 100mg PCL
PC75/PCL25	150mg BPA : 150mg DPC : 3mg NaOH : 100mg PCL
PC67/PCL33	100mg BPA : 100mg DPC : 2mg NaOH : 100mg PCL
PCL	PCL only

### 2.4. Impregnation of PC/PCL Blend

Sample PC67/PCL33 (Table 1) underwent an impregnation with ibuprofen, followed by a dissolution study. The PC67/PCL33 sample was placed in a 5ml beaker and loaded alongside another beaker with excess ibuprofen inside a 100 mL temperature-controlled stainless steel reactor. The 4 hr experiment was conducted at 40 °C and 200 bar. Also, a control experiment with PCL from Sigma was conducted.

### 2.5. Analytical Techniques

#### Gel Permeation Chromatography

Gel permeation chromatograms were obtained using a Shimadzu GPC with tetrahydrofuran (THF) as the mobile phase. The GPC was calibrated using PS standards. Mark-Houwink parameters (K = 40 x $10^{3}$, α=0.7) were used to relate the viscosity of PC in THF to the PS standards used for THF GPC calibration [31].

#### Differential Scanning Calorimetry

A TA Instruments DSC 2010 Differential Scanning Calorimeter was used with N${}_{2}$ gas flow, and a gradient of 20 ${\;}^{\circ }C$/min from -100 ${\;}^{\circ }C$ up to 200 ${\;}^{\circ }C$.

#### Scanning Electron Microscopy

A Hitachi S3400i was used to take images of the PC/PCL blends (Table 1).

#### Nitrogen Adsorption

Porosity analysis of the PC/PCL blends (Table 1) was conducted on a Micromeritics TriStar 3000 Analyzer using nitrogen adsorption.

#### Reverse-phase High Performance Liquid Chromatography

Reverse-phase high performance liquid chromatography was used to quantify the drug-loading of ibuprofen. The PC monomers had a similar UV absorbance to the ibuprofen, therefore a RP-HPLC method was developed to separate the compounds. A RP-HPLC method was developed on a Waters system which included a 717 plus autosampler and a 515 HPLC pump. The system was equipped with a Waters 996 photodiode array detector. A Lichrosorb RP18 analytical column from Phase Separations was housed in an Activon column oven at 60 ${\;}^{\circ }C$. The starting mobile phase was 10% THF/90% deionized water at a flow-rate of 1.5 mL/min. After 150 min the mobile phase was changed to 50% THF/50% deionized water over a period of 15 min. The RP-HPLC system was also used to construct a calibration curve for ibuprofen. The calibration was obtained using an isocratic mobile phase of 10% THF/90% deionized water.

#### Dissolution Study

Powder dissolution studies were performed under sink conditions for ibuprofen. The dissolution studies used the paddle method in 100 mL deionized water at 37 ${\;}^{\circ }C$ and stirring at 50 rpm. Aliquots of 4 mL were withdrawn at specific time intervals. The concentration of ibuprofen was quantified using a UV spectrophotometer at an absorbance of 220 nm.

## 3. Results and Discussion

### 3.1. PC Synthesis

As reported in Table 2, experiments conducted at 200 ${\;}^{\circ }C$ did not produce higher M${}_{w}$ PC than the experiments conducted at 120 ${\;}^{\circ }C$ and 150 ${\;}^{\circ }C$. None of the PC prepolymer synthesized in N${}_{2}$ exceeded a M${}_{w}$ of 1.5 x 10${}^{3}$ Da. The experiments under N${}_{2}$ were conducted in a isolated system resulting in the accumulation of phenol, which in turn slowed down the generation of higher M${}_{w}$ PC. The experiments conducted at 120 ${\;}^{\circ }C$ produced the highest M${}_{w}$. Of the tested conditions (Table 2), 120 ${\;}^{\circ }C$ was the most favourable operating condition, whilst increasing the reaction time over hours did not improve the M${}_{w}$.

Comparison between the M${}_{w}$ of samples PC-6 hrs (3.9 x 10${}^{3}$ Da) from Table 6 to the M${}_{w}$ of sample N${}_{2}$-2-CO${}_{2}$-4-120 (2.56 x 10${}^{3}$ Da) from Table 5 shows the effectiveness of the three-stage polymer synthesis method over the two-stage methods. The use of a multi-stage PC synthesis method was derived from the aforementioned results and results reported by Kiserow and co-workers [5,32], and Lee *et al.* [6]. Results from Table 4, 5 and 6 reflect the effectiveness of a multi-stage prepolymer synthesis approach for 10% w/w catalyst to monomers, with varying temperature, dense CO${}_{2}$ pressure and a set dense CO${}_{2}$ flow-rate of 0.3–0.5 mL/min.

**Table 2:** PC synthesis in N${}_{2}$.

Sample	Time	Temp	M${}_{w}$x10${}^{3}$	BPA Conv	DPC Conv
	(hr)	(${\;}^{\circ }C$)	(Da)	(%)	(%)
N${}_{2}$-1-120	1	120	1.18	78.3	81.3
N${}_{2}$-4-120	4	120	1.26	79.3	81.9
N${}_{2}$-12-120	12	120	1.20	79.5	85.8
N${}_{2}$-1-150	1	150	0.90	80.1	82.4
N${}_{2}$-4-150	4	150	0.99	98.9	99.4
N${}_{2}$-8-150	8	150	0.92	98.9	99.4
N${}_{2}$-24-150	24	150	1.00	82.0	N/A
N${}_{2}$-48-150	48	150	0.90	86.0	89.8
N${}_{2}$-1-200	1	200	0.75	81.0	N/A
N${}_{2}$-4-200	4	200	0.78	84.9	99.7
N${}_{2}$-48-200	48	200	0.90	86.3	99.9

The three stages 6 hr PC synthesis method produced a PC with a M${}_{w}$ of 3.9 x 10 ${}^{3}$ Da. The third stage 150 ${\;}^{\circ }C$, 200 bar with a dense CO${}_{2}$ flux has shown to increase the M${}_{w}$ when compared to the two-stage process N${}_{2}$-2-CO${}_{2}$-4-120 (M${}_{w}$ = 2.56 x 10${}^{3}$ Da). Results with this work are in line with both Lee *et al.* [6] and Kiserow and co-workers [5,32] on the effectiveness of phenol extraction by dense CO${}_{2}$.

The PC synthesis in this work has produced a polymer with M${}_{w}$ comparable to other dense gas processes. The three-stage PC synthesis procedure used in this work used a lower amount of catalyst and organic solvents than conventional methods.

**Table 3:** M${}_{w}$ based on GPC and ESI-MS.

Sample	GPC	ESI-MS
	M${}_{w}$ x 10${}^{3}$ (Da)	M${}_{w}$ x 10${}^{3}$ (Da)
N${}_{2}$-24-150	1.00	0.94
N${}_{2}$-48-150	0.90	0.92
N${}_{2}$-1-200	0.75	0.87

**Table 4:** GPC results for PC synthesis in N${}_{2}$ and CO${}_{2}$ at 150 ${\;}^{\circ }C$. Two-stage process.

Sample	Stage 1 time	Stage 2 time	Total	M${}_{w}$x10${}^{3}$
	N${}_{2}$(hrs)${}^{†}$	CO${}_{2}$(hrs)${}^{♮}$	(hrs)	(Da)
N${}_{2}$-2-CO${}_{2}$-2	2	2	4	1.40
N${}_{2}$-4	4	0	0	0.99
N${}_{2}$-4-CO${}_{2}$-1	4	1	5	1.33
N${}_{2}$-4-CO${}_{2}$-4	4	4	8	2.60
N${}_{2}$-4-CO${}_{2}$-8	4	8	12	1.48

${}^{†}$ = isolated at 1 bar

${}^{♮}$ = 100 bar with a flow-rate of 2 mL/min

**Table 5:** GPC results for PC synthesis in N${}_{2}$ and CO${}_{2}$ at 120 ${\;}^{\circ }C$. Two-stage process.

Sample	Stage 1 time	Stage 2 time	Total	M${}_{w}$x10${}^{3}$
	(hrs)${}^{†}$	(hrs)${}^{‡}$	(hrs)	(Da)
N${}_{2}$-2-120	2	0	2	1.28
N${}_{2}$-2-CO${}_{2}$-2-120	2	2	4	1.82
N${}_{2}$-2-CO${}_{2}$-4-120	2	4	6	2.56
N${}_{2}$-4-CO${}_{2}$-8-120	2	8	10	4.73

${}^{†}$ = isolated at 1 bar

${}^{‡}$ = 200 bar with a flow-rate of 0.3–0.5 mL/min

**Table 6:** GPC results for the three-stage PC synthesis at 120 ${\;}^{\circ }C$.

Sample	Stage 1 (hrs)	Stage 2 (hrs)	Stage 3 (hrs)	M${}_{w}$x10${}^{3}$ (Da)
PC-6hrs	2	2	2	3.9
PC-8hrs	4	2	2	3.8
PC-8hrs-stage3	2	2	4	4.7

### 3.2. Synthesis of PC with Dense CO${}_{2}$-foamed PCL

**Figure 4:**
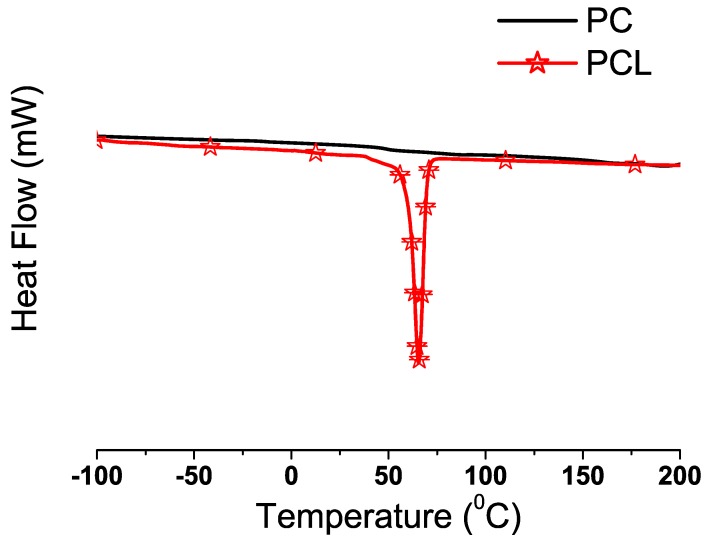
DSC profiles of PC and PCL (exothermic up).

**Figure 5:**
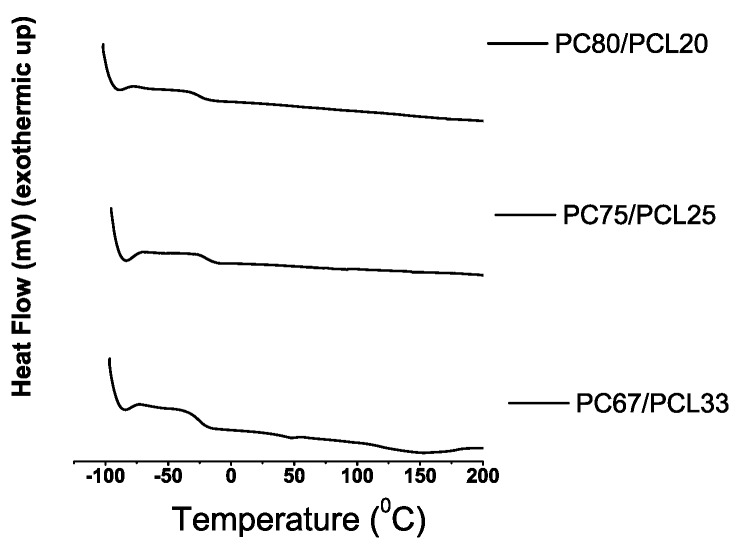
DSC profiles of PC/PCL blends.

The PC/PCL DSC profiles and their corresponding T${}_{g}$ values can be found in Figure 5 and Table 7, respectively. Within the depicted temperature range, only PC67/PCL33 had a broad endothermic transition characteristic of a T${}_{m}$ at 155${\;}^{\circ }C$. The T${}_{g}$ values for the PC/PCL blends are all similar, and higher than the T${}_{g}$ of PCL (-60 ${\;}^{\circ }C$) (Figure 4). The composition-dependent shifting of a T${}_{g}$ correspond to samples with intimate level of blending. The T${}_{m}$ of PCL at 60 ${\;}^{\circ }C$ and T${}_{g}$ of PC at 145 ${\;}^{\circ }C$ were not observed in the DSC profiles of the blends. The DSC profiles of all the samples (Figure 5) support the existence of a strong interaction between PC and PCL. In particular, the absence of T${}_{m}$ and the shifting of the T${}_{g}$ indicates intimate and molecular blending [33].

**Table 7:** T${}_{g}$ for the PC/PCL samples.

Sample	T${}_{g}$ (${\;}^{\circ }C$)
PC80/PCL20	−31
PC75/PCL25	−26
PC67/PCL33	−31

#### Morphology

**Figure 6:**
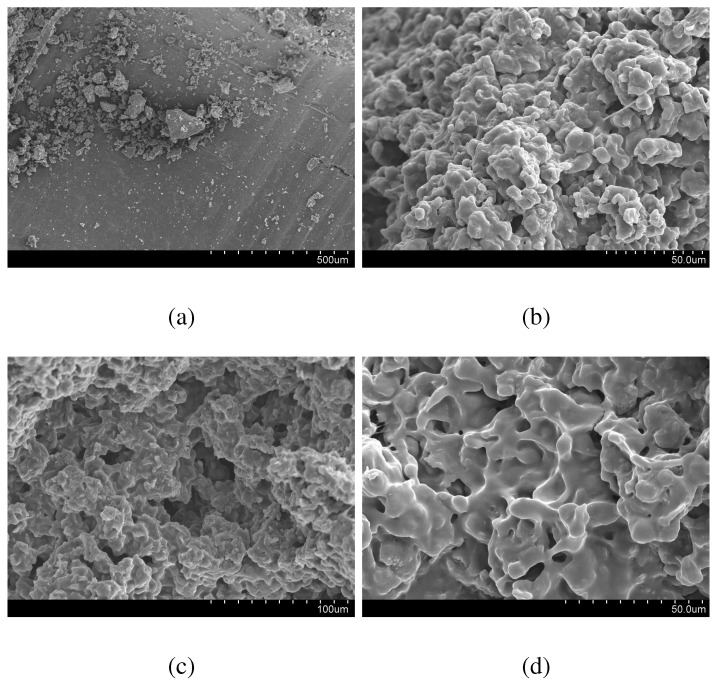
SEM image of (a) PC, (b) PC80/PCL20, (c) PC75/PCL25, (d) PC67/PCL33.

The SEM image of neat PC did not exhibit any porosity, whilst the PC/PCL samples were porous (Figure 6). Interconnected micro-pores were observed in the SEM images of the PC/PCL blends. The ability of CO${}_{2}$ to form pores and foam PC has been documented in the scientific literature [34,35,36]. Previously documented cases involved longer processing times, higher pressures and temperatures [34,35,36]. The formation of pores in the PC/PCL blends most likely occurred because of the ability of PCL to foam in the presence of CO${}_{2}$. From the SEM images of the PC/PCL blend surfaces it is evident that the sizes of the pores are in the micron range.

**Figure 7:**
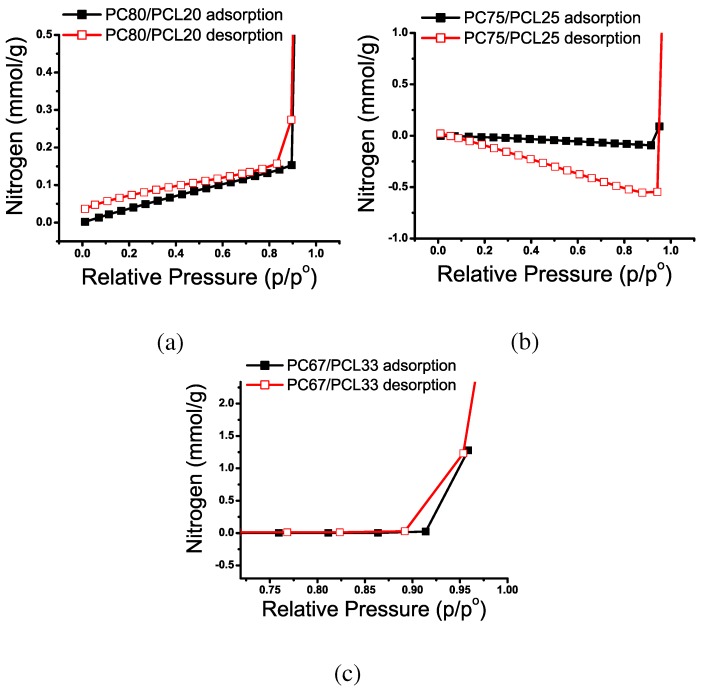
BET isotherms: (a)PC80/PCL20 (b)PC75/PCL25 (c)PC67/PCL33.

According to the International Union of Pure and Applied Chemistry (IUPAC), pores are classified into three ranges based on internal diameter; micropores, mesopores and macropores (Table 9) [37,38]. Each type of internal pore width exhibits a characteristic adsorption pattern. Also, IUPAC has classified the sorption isotherms into six groups based on their sorption patterns (Figure 9 [38]). Each of the adsorption isotherms in Figure 7 exhibited Type II sorption behaviour, which is typical of a macroporous adsorbent. Each PC/PCL adsorption isotherm exhibited some form of hysteresis. Hysteresis indicates that the adsorption and desorption of N${}_{2}$ molecules followed different pathways, and were possibly obstructed by the existence of interconnected pores. From the adsorption isotherms it is difficult to confidently quantify a pore width. However by analyzing the adsorption profile and comparing it against the IUPAC classified isotherms, one can confidently identify micropores, mesopores or macropores. Nitrogen adsorption is most accurate for quantifying pore sizes less than 300 nm, and the SEM images (Figure 6) clearly indicate the existence of pores larger than 300 nm. Also the adsorption/desorption isotherms of the PC/PCL blends clearly indicate a high incidence of macropores.

### 3.3. Drug Loading and Dissolution

Blend PC67/PCL33 (Table 1) was chosen as a candidate for drug-loading because of its porous morphology, morphology that was ideal for tissue engineering scaffold-type applications. The PC67/PCL33 blend had the highest fraction of PCL, and therefore the greatest potential for higher drug-loading compared to the PC80/PCL20 and PC75/PCL25.

The PCL component provided a pathway for the dense CO${}_{2}$ impregnation of ibuprofen. Ibuprofen is a CO${}_{2}$-soluble therapeutic agent [39]. Both the blend and a control sample of PCL were impregnated at 40 ${\;}^{\circ }C$ and 200 bar in 4 hrs, and drug loadings of 9 wt% and 20 wt% (Table 8) were observed. Polycaprolactone is susceptible to melting point depression in the presence of dense CO${}_{2}$ [40]. Despite the lower PCL content, the PC67/PCL33 had a higher drug-loading than pure PCL, which may be correlated to both the higher porosity and bumpy surface morphology (Figure 6). The pores may have provided a means for the solubilized ibuprofen to travel deeply into the PC/PCL matrix which had a larger surface area to adsorb onto.

**Table 8:** Drug loading after impregnation.

Sample	Drug Loading (wt%)
PCL	9
PC67/PCL33	20

**Table 9:** IUPAC classification of internal pore width.

Pore Classification	Definition
Micropore	internal width < 2 nm
Mesopore	2 nm < internal width < 50 nm
Macropore	internal width > 50 nm

**Figure 8:**
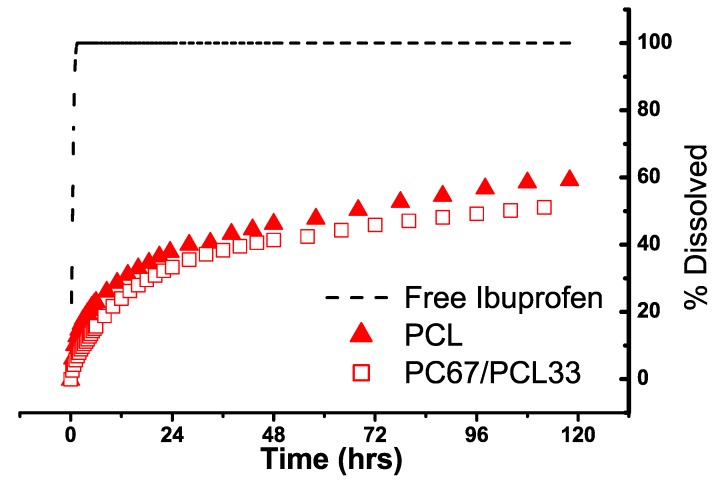
Dissolution profiles of free ibuprofen, PCL and PC67/PCL33.

A dissolution study was conducted to monitor the release profile of ibuprofen from the impregnated samples. The free ibuprofen dissolved completely in minutes (Figure 8), whereas the ibuprofen in the PCL and PC/PCL blend displayed a sustained release over days (Figure 8). After 5 days, approximately 60% and 50% of drug was released from PCL and PC67/PCL33 respectively. Both PCL and PC67/PCL33 exhibited first order dissolution profiles. In summary, the PC/PCL blends exhibited porosity and sustained drug release, thereby making them potential candidates for biomedical applications such as drug delivery devices and tissue scaffolding.

**Figure 9:**
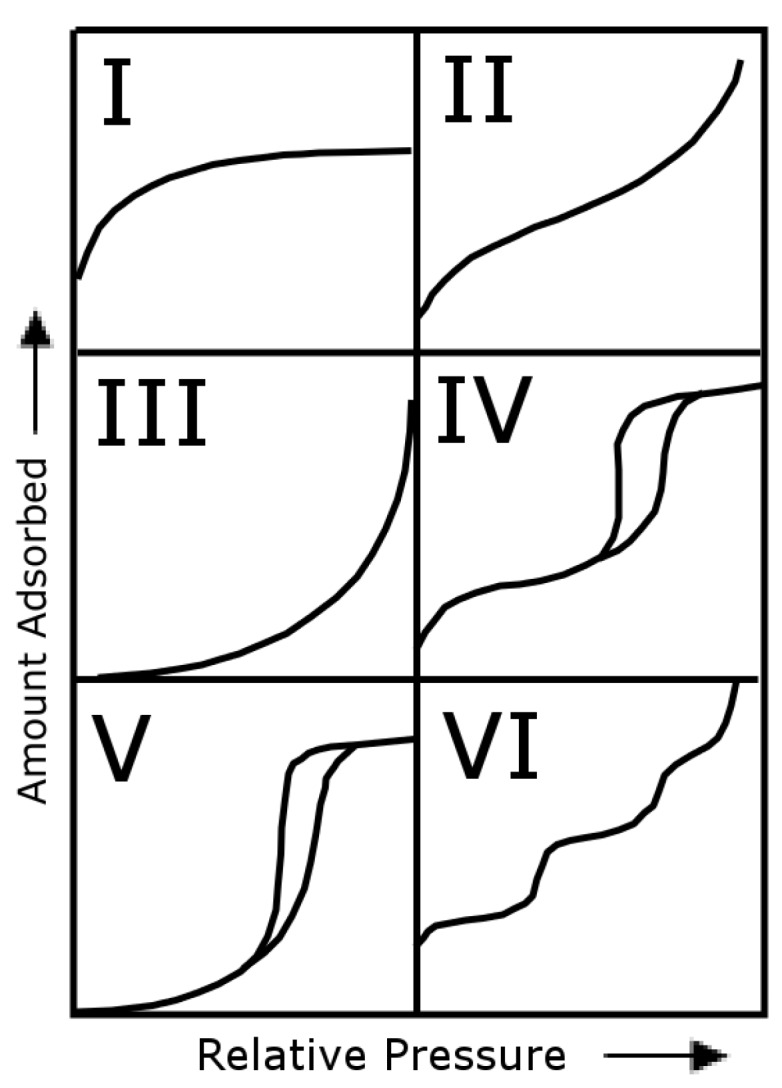
IUPAC classification of sorption isotherms. I = microporous, II = macroporous, III = non-porous or microporous, IV = mesoporous, V = microporous/mesoporous, VI = non-porous.

## 4. Conclusion

The use of dense CO${}_{2}$ as a polymerization medium to synthesize PC, and then as a processing medium to create PC/PCL blends was proven to be successful in creating intimate bio-blends. A unique dense CO${}_{2}$ blending technique was used; the synthesis of PC in the presence of dense CO${}_{2}$-swollen PCL particles.

The three-stage melt-phase polymerization/blending method proved to be an advantageous use of dense CO${}_{2}$ for the production of macroporous PC/PCL blends. A macroporous architecture was observed for PC/PCL mass ratios of 80/20, 75/25 and 67/33. The DG synthesis of PC in the presence of PCL particles has proven to work as a blending technique, and selectively permits the synthesis of PC whilst extracting the phenol. Compared to already existing blending methods, DGT provides a more environmentally benign method void of organic solvents, and an alternative route to pore-formation void of toxic porogens.

Bio-blends of PC/PCL have potential industrial and biomedical applications. The biodegradability of PCL has long been exploited in drug delivery devices. In this work, ibuprofen, an anti-inflammatory agent was successfully impregnated into a macroporous PC/PCL blend. A drug-loading of 20 wt% was obtained using DGT. Also, a sustained release profile was observed over 5 days. Mechanical testing is advisable to further prove the industrial and biomedical applicability of DG produced PC/PCL blends.
